# Blount Disease

**DOI:** 10.5334/jbsr.2557

**Published:** 2021-09-23

**Authors:** Sophia Chkili, Paolo Simoni

**Affiliations:** 1 “Reine Fabiola” Children’s University Hospital, BE

**Keywords:** Blount disease, Genu Varum, Magnetic Resonance Imaging, Radiography/diagnosis

## Abstract

**Teaching point**: MRI allows to assess deformity and viability of the tibia in Blount disease.

## Case Presentation

A seven-year-old girl was referred because of bilateral genu varum that was more apparent on her right side. The rest of the clinical examination was unremarkable, apart from a slight overweight. A small protuberance was palpated at the medial proximal metaphysis of her right tibia.

**Figure 1:**
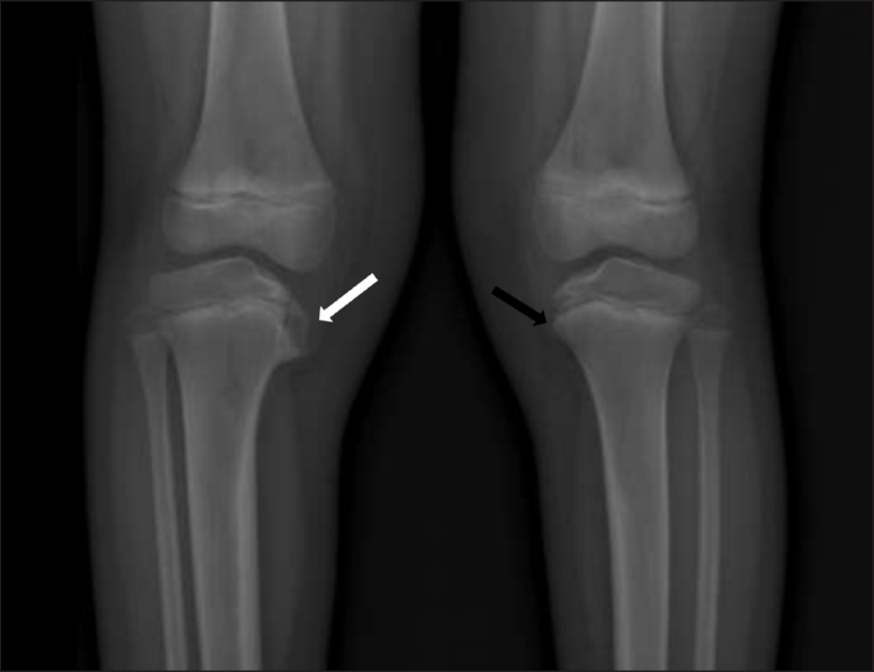


Conventional anteroposterior radiographs of knees in upstanding position showed a “beak-like” protuberance of the medial tibial plateau of the right side (***Figure 1***, white arrow) and a subtle abnormality of the corresponding region of the other side (***Figure 1***, black arrow).

**Figure 2:**
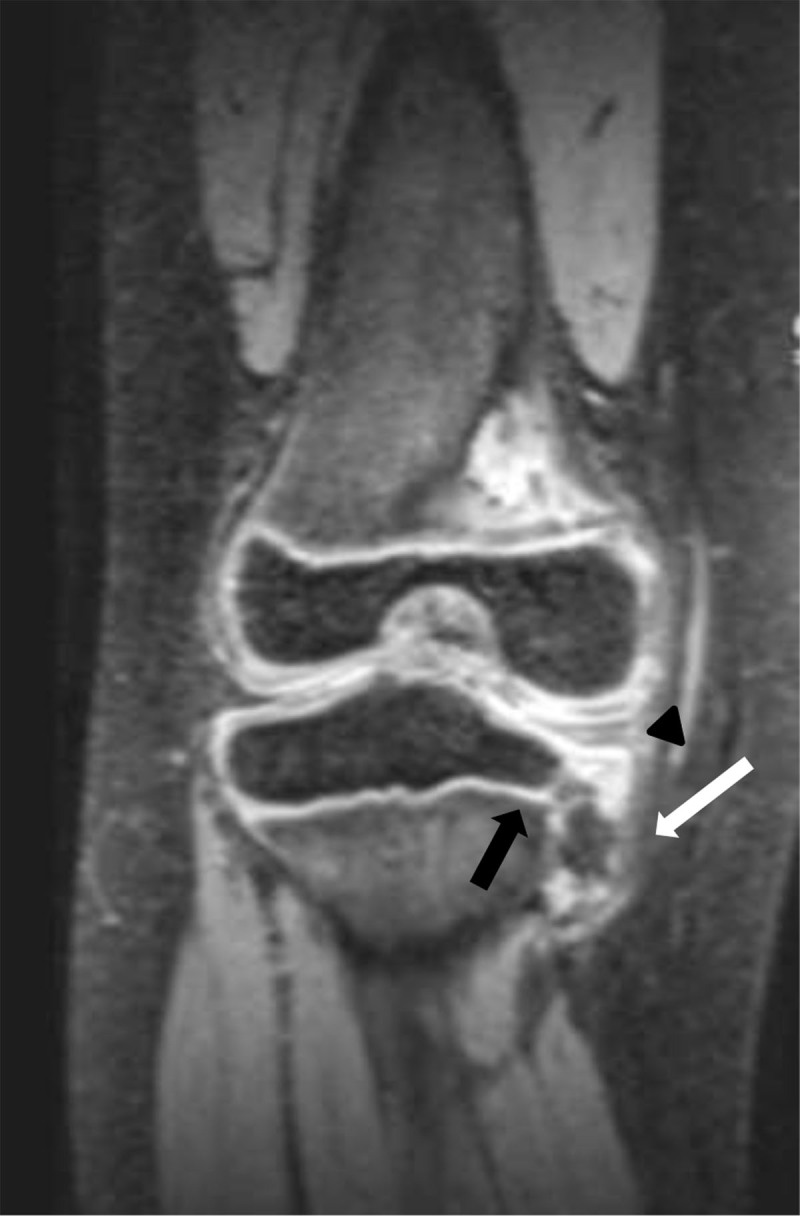


Proton density-weighted magnetic resonance imaging (MRI) on coronal plane (***Figure 2***) showed an abnormal osteochondral maturation of the proximal medial tibial plateau and the adjacent metaphysis. Epiphyseal and metaphyseal cartilage last unossified and distorted. Islands of anarchic bone ossification (***Figure 2***, white arrow) corresponds to the “beak-like appearance” visible on the radiograph. The growth plate appeared normal on MRI except for its medial third on the medial tibial plateau, which was slightly concave and thinned. Medial femoro-tibial joint space was enlarged (***Figure 2***, black arrowhead). The interposed medial meniscus seemed thickened and globally of high signal with no visible lesion.

## Discussion

Blount disease is a disorder of proximal tibial growth that produces a three-dimensional deformity [1]. Patients often present with genu varum.

Two clinical presentations were described: infantile or early-onset form and adolescent (also called late-onset) form. The cut-off age to distinguish the two presentations is ten years of age. Blount disease is initially bilateral in 50% of cases [1].

Blount disease have been linked to obesity and overweight. A predominance in African American boys was also reported [1].

The pathogenesis of Blount disease is still unclear. Vascular failure during the epiphyseal growth is advocated as a cause of the growth disturbance of the proximal medial tibia.

**Figure 3:**
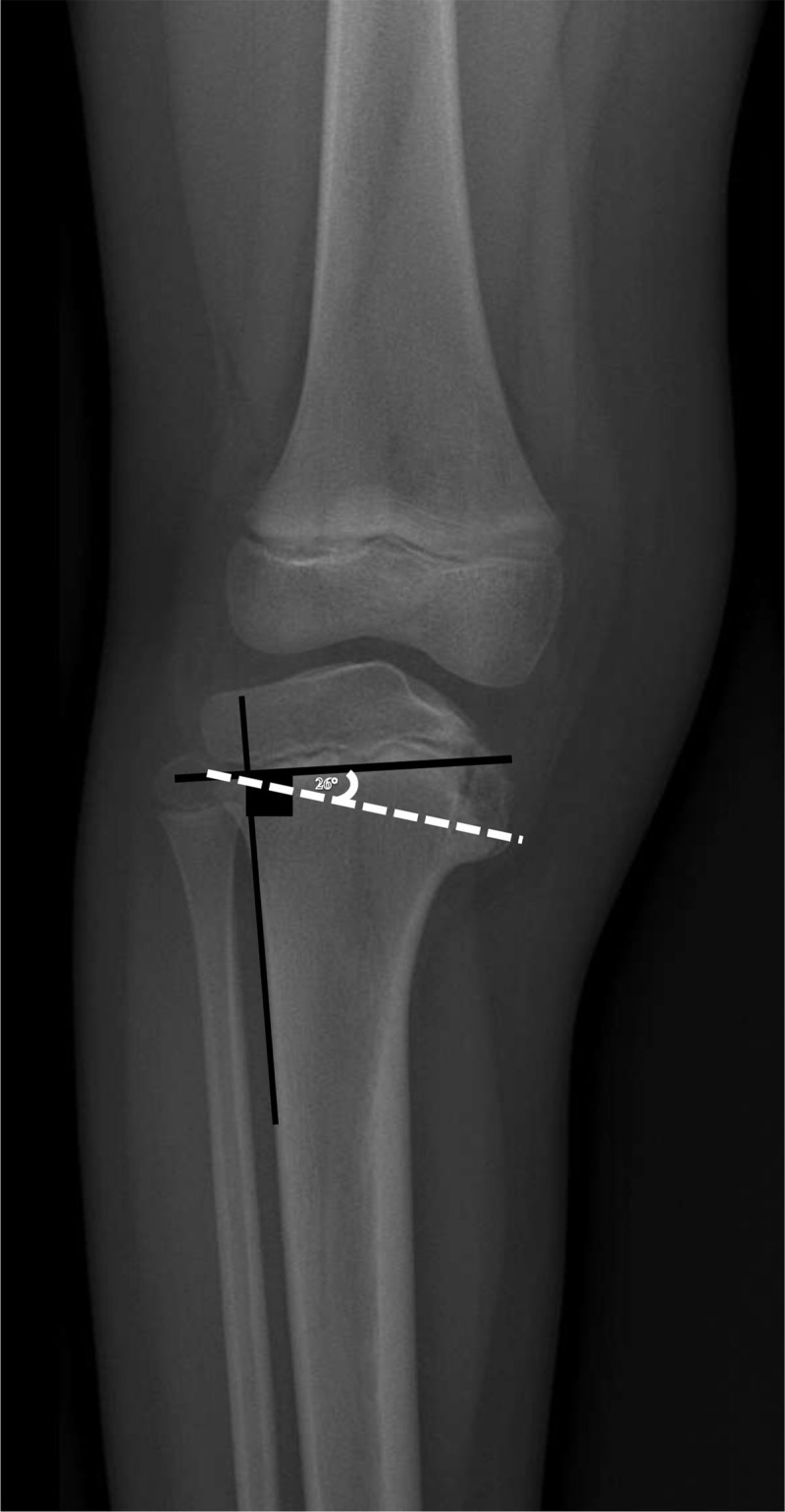


Radiograph shows the protuberance to evaluate the slope of the medial tibial plateau. The most used measurement is the metaphyseal-diaphyseal angle (MDA). MDA is measured by determining the angle between the line through the two beaks of the metaphysis and the line perpendicular to the lateral aspect of the tibial diaphysis (***Figure 3***). MDA higher than 16° is considered abnormal. When the MDA is between 11° and 15°, Blount disease should be ruled out based on bone appearance.

Besides the visualization of cartilage and menisci, MRI allows a three-dimensional assessment of the deformity. Some studies suggested that dynamic contrast-enhanced MRI could detect irreversible alterations of the physis. Untreated Blount disease is associated with a poor prognosis, especially in young children due to the growth spurt and the early fusion of the growth plate occurring from the ages of six to eight [1].
